# Representational Switching by Dynamical Reorganization of Attractor Structure in a Network Model of the Prefrontal Cortex

**DOI:** 10.1371/journal.pcbi.1002266

**Published:** 2011-11-10

**Authors:** Yuichi Katori, Kazuhiro Sakamoto, Naohiro Saito, Jun Tanji, Hajime Mushiake, Kazuyuki Aihara

**Affiliations:** 1 FIRST, Aihara Innovative Mathematical Modelling Project, JST, Kawaguchi, Japan; 2 Collaborative Research Center for Innovative Mathematical Modelling, Institute of Industrial Science, The University of Tokyo, Tokyo, Japan; 3 Research Institute of Electrical Communication, Tohoku University, Sendai, Japan; 4 Department of Physiology, Tohoku University School of Medicine, Sendai, Japan; 5 CREST, JST, Kawaguchi, Japan; Indiana University, United States of America

## Abstract

The prefrontal cortex (PFC) plays a crucial role in flexible cognitive behavior by representing task relevant information with its working memory. The working memory with sustained neural activity is described as a neural dynamical system composed of multiple attractors, each attractor of which corresponds to an active state of a cell assembly, representing a fragment of information. Recent studies have revealed that the PFC not only represents multiple sets of information but also switches multiple representations and transforms a set of information to another set depending on a given task context. This representational switching between different sets of information is possibly generated endogenously by flexible network dynamics but details of underlying mechanisms are unclear. Here we propose a dynamically reorganizable attractor network model based on certain internal changes in synaptic connectivity, or short-term plasticity. We construct a network model based on a spiking neuron model with dynamical synapses, which can qualitatively reproduce experimentally demonstrated representational switching in the PFC when a monkey was performing a goal-oriented action-planning task. The model holds multiple sets of information that are required for action planning before and after representational switching by reconfiguration of functional cell assemblies. Furthermore, we analyzed population dynamics of this model with a mean field model and show that the changes in cell assemblies' configuration correspond to those in attractor structure that can be viewed as a bifurcation process of the dynamical system. This dynamical reorganization of a neural network could be a key to uncovering the mechanism of flexible information processing in the PFC.

## Author Summary

The prefrontal cortex plays a highly flexible role in various cognitive tasks e.g., decision making and action planning. Neurons in the prefrontal cortex exhibit flexible representation or selectivity for task relevant information and are involved in working memory with sustained activity, which can be modeled as attractor dynamics. Moreover, recent experiments revealed that prefrontal neurons not only represent parametric or discrete sets of information but also switch the representation and transform a set of information to another set in order to match the context of the required task. However, underlying mechanisms of this flexible representational switching are unknown. Here we propose a dynamically reorganizable attractor network model in which short-term modulation of the synaptic connections reconfigures the structure of neural attractors by assembly and disassembly of a network of cells to produce flexible attractor dynamics. On the basis of computer simulation as well as theoretical analysis, we showed that this model reproduced experimentally demonstrated representational switching, and that switching on certain characteristic axes defining neural dynamics well describes the essence of the representational switching. This model has the potential to provide unique insights about the flexible information representations and processing in the cortical network.

## Introduction

The prefrontal cortex (PFC) is believed to play crucial roles in flexible decision making and action planning that are essential for adapting to an ever-changing real world. Prefrontal neurons hold not only multiple sets of discrete information and parametric magnitudes of stimuli in their working memory but also transform online information to behaviorally relevant information that is required under a given behavioral context [1], [2], [3], [4], [5], [6], [7]. Such “representational switching” is observed in PFC neurons when subjects are undertaking various cognitive tasks, e.g., “what–where” working-memory tasks [7], location–object comparison tasks [6], two-interval discrimination tasks [5], duration-discrimination tasks [1], and goal-oriented action-planning tasks [2], [3], [4]. These tasks usually require the holding of information as working memory during delay periods and the appropriate processing of information to guide behavior in a given context. For example, in the goal-oriented action-planning task, many prefrontal neurons initially encode a behavioral goal and then a part of these neurons subsequently encodes a future action [2], [4]. This dynamical encoding by prefrontal neurons can be interpreted as the switching of mapping between patterns of neural activity and sets of information. We assume that a set of information (e.g., a set of goals or a set of actions) is mapped onto an ensemble of neurons. Initially, one functional mapping may be manifested in local circuits and adaptively switched to another functional mapping toward the end of delay periods of the task. The PFC is seated on the highest level of a functional hierarchy of the sensation-action process and represents abstract aspects of complex sensory and action information [8]. The PFC contributes to planning and generation of actions with its internal dynamics, rather than with mere stimulus-response associations [9]. This ubiquitous adaptability to different functions in various tasks, which has been revealed by both electrophysiological and imaging studies, suggests that the mechanism of adaptive neural coding in the PFC may be general. However, little is known about the mechanism. In this study, we investigate the mechanism of representational switching by using a computational model of a prefrontal neural network.

The abovementioned tasks require the storage of information in a delay period of a given task by using the working memory that is realized with sustained neural activity [10], [11]. Stably sustained neural activity can be theoretically characterized by attractor dynamics [12] with a feedback mechanisms [13], [14], [15]. In a conventional attractor network, there generally exist multiple attractors, each of which distinguishes one discrete set of categories or information and shifts to another attractor by external inputs or noise depending on the required task [16], [17], [18]. However, such a state transition does not change the structure of the attractors in the state space that reflects the mapping between the attractors and information. Recent studies show, on the other hand, the possibility that the generation of representations is embedded in sequentially changing cell assemblies with the modulation of synaptic efficacy [19], [20], [21]; however, the underlying theoretical mechanism and the roles of this dynamical reorganization of cell assemblies in representational switching is still unclear.

**Figure 1:**
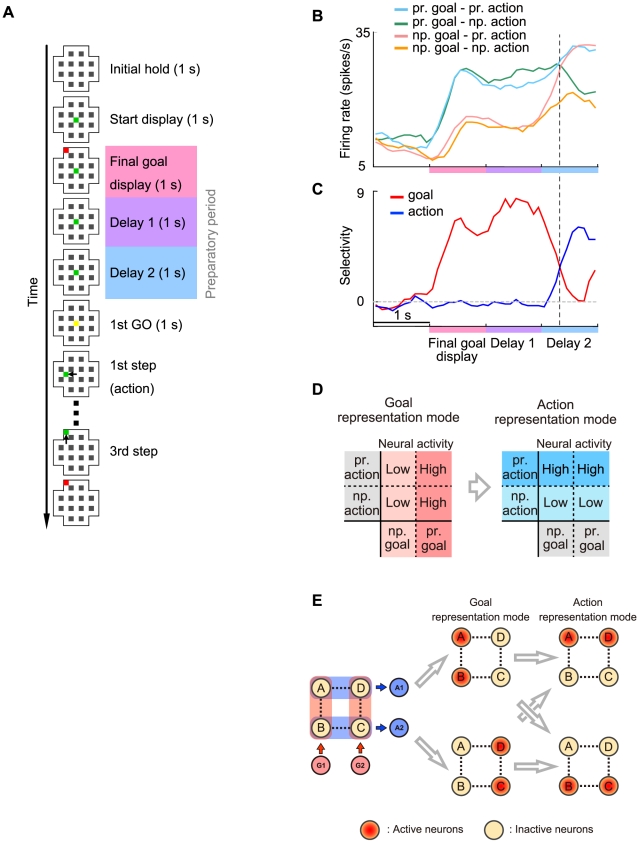
Goal-oriented action-planning task and neuronal responses in prefrontal cortex (PFC). (**A**) Temporal sequence of events in the task [2], [3], [4]. Each panel represents a maze that was displayed on a monitor. Green squares indicate current cursor positions and red squares indicate goal positions. The yellow square represents the movement initiation signal. Short black arrows indicate cursor movements. Red and blue/purple panels indicate the goal-display period and the preparatory period, respectively. (**B**) Discharge properties of a PFC neuron that first represents the goal position followed by the action direction. The spike-density histograms of neuronal activity under task conditions are indicated by colored curves, showing each combination of preferred (pr.) and non-preferred (np.) goal positions and action directions. (**C**) Time course of goal-position and action-direction selectivity measures. The regression coefficient, calculated from the histograms in (**B**), is normalized and plotted for the goal position (red) and the action direction (blue). (**D**) Properties of neural responses before and after the representational switching. In the goal (action) representation mode, the activity of the neuron becomes high for the preferred goal (action), while it becomes low for the non-preferred goal (action). Each neuron has the selectivity for one goal position and one action direction. (E) Inferred changes of possible patterns of neural activity in the situation with two goals and two actions.

In the present study, we propose a switching network model based on the dynamical reorganization of attractor structure by internal changes in synaptic connectivity. In particular we used short-term synaptic plasticity [22], [23], [24], [25], [26] as a component of the model network for representational switching because synapses with short-term plasticity can facilitate global reorganization of functional cell assemblies in networks. Furthermore, because of gradual changes in synaptic efficacy, this network is able to hold one set of information before representational switching and another different set afterward, and endogenously generate representational switching of neuronal activity in a flexible manner. More specifically, we have developed a mathematical model of a lateral PFC network that performs the goal-oriented action-planning task (Figure 1A). In this task, the PFC encodes dual information: goal positions and action directions as firing rates of prefrontal neurons [2], [3], [4]. Representations of the two different categories of information coexist and are endogenously transformed in the middle of the delay period [2], [4]. The present model qualitatively reproduces these experimentally demonstrated responses in the PFC.

## Results

### Representational Switching in Lateral PFC during Goal-Oriented Action-Planning Task

Figure 1 shows the temporal sequence of the goal-oriented action-planning task [2], [3], [4] and the responses of a lateral PFC neuron that were recorded from a monkey who was trained to move the cursor on the screen to a goal presented during a goal-display period. Neural activity depends on the phase of the task, the position of the goal, and the direction of the action. After the cursor is displayed at the start position, the final goal position is displayed during the goal-display period. If a prefrontal neuron prefers the displayed goal position, the firing rate of the neuron increases compared to that in the case when a non-preferred goal was displayed (Figure 1B). Although the display of the goal position disappears in the next delay period, the goal-position-related activity in the prefrontal neuron persists until the middle of the delay period. It should be noted that although some neurons show goal-position-related activity in the entire period of the task, we focus here on such neurons that show representational switching from the goal mode to the action mode [2], [4].

After the delay period, the first movement-initiation signal (the “Go” signal) appears, and the monkey is required to move the cursor stepwise to reach the goal. In this task, the neuron showed representational switching in the middle of the delay period and persistent activity depending on the action direction during the remaining delay period. Note that the representational switching precedes the Go signal [2], [4], suggesting that the representational switching is not triggered by an external signal or a sensory cue. Representational switching is more clearly shown by the selectivity measure (Figure 1C), which is obtained by multiple linear regression analysis [4], [27] (see Methods for details). The selectivity measure indicates the switching from the goal-representation mode to the action-representation mode.

Each neuron involved in the representational switching has a preference for both of a goal position and an action direction as shown in Figure 1D. In the goal representation mode, the activity of the neuron becomes high for the preferred goal, while it becomes low for the non-preferred goal. In the action representation mode, on the other hand, the neural activity becomes high for the preferred action direction, while it becomes low for the non-preferred action.

What can neural mechanisms be inferred from this result? Each neuron shows large responses for one of goals and one of actions, and switches its responsibility from the goal representation mode to the action representation mode in the middle of the delay period. For simplicity, suppose that two goals and two actions are involved in this task (see Figure 1E). Possible patterns of neural activity in each representation mode are limited as in Figure 1E. In the goal (action) representation mode, possible combinations of sustained neural activity states are A&B or C&D (A&D or B&C).

Considering mutual connections between simultaneously activated neurons, the sustained neural activity in a specific group of neurons is also understandable with the conventional attractor framework. Mutually connected neurons form a cell assembly and the active state corresponds to an attractor. However, it is puzzling how the network dynamically reconfigures patterns of neural activity and switches representation modes. It is a natural idea that external stimuli trigger the transition among attractors, but if so, then an equivalently difficult problem of how such stimuli are generated by neural networks remains as unsolved.

### Conceptual Model of Dynamically Reorganizable Attractor Network

**Figure 2:**
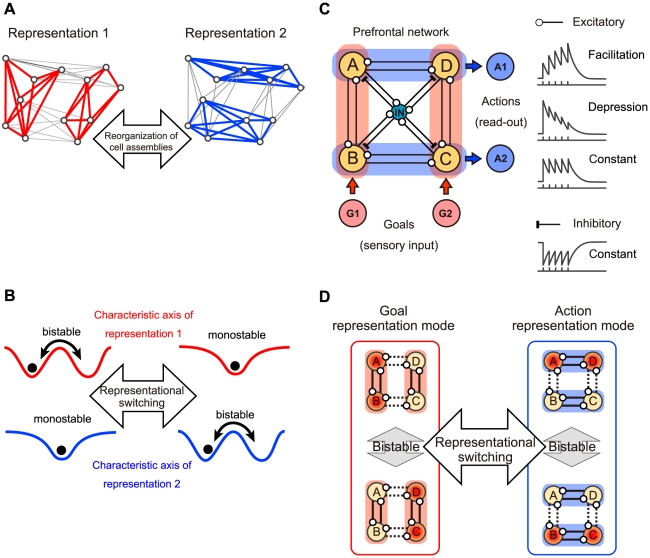
Dynamically reorganizable attractor network model. (**A, B**) Conceptual diagrams of representational switching in a network that represents multiple sets of information. Its representation modes are dynamically switched because of changes in synaptic connectivity. (**A**) The cell assemblies that are formed with relatively strong connections, indicated by red and blue lines. (**B**) Schematic landscapes of attractors in the characteristic axes that define the dynamics of each representation mode. If the stability along a characteristic axis is bistable with double wells, the network is able to represent two fragments of information. However, if the stability is monostable with a single well, the network is unable to represent this information (see Figure S1). (**C**) Network structure of the model. Each node of A, B, C, and D represents a population of excitatory neurons in the prefrontal cortex. The smaller blue node (IN) represents a population of inhibitory interneurons. Node G1 (G2) is a population of neurons that represents goal position 1 (goal position 2) and sends excitatory inputs to nodes A and B (C and D). A1 (A2) is a population of motor neurons representing action direction 1 (action direction 2) and read-out activity of nodes A and D (B and C). (**C** right) Schematic time courses of synaptic activities. Connections among population A, B, C, and D with a circle indicate the synapses which can be accompanied with facilitation or depression. The synaptic connections between excitatory and inhibitory interneurons are assumed to be without any short-term plasticity for simplicity (see Figure S2 for possible network structure for a higher-dimensional task). (**D**) Possible states in the model. Red (yellow) nodes indicate the active (inactive) state of the neural populations. The relatively strong connections are indicated by solid lines, and form cell assemblies. The short-term changes in synaptic connectivity result in reorganization of the cell assemblies and subsequent representational switching.

We propose a neural network model in which cell assemblies [19], [28] that encode fragments of information are functionally structured and the formation of cell assemblies can be dynamically updated through the modulation of synaptic connections (Figure 2A). The activity of the cell assemblies is triggered or read out by other networks that encode specific input or output information. If two sets of information such as goals and actions are represented in the neural network, the network can be characterized with two different formations of cell assemblies before and after representational switching. Each formation of cell assemblies corresponds to the formation of attractors that can be described on two characteristic axes (Figure 2B). Each characteristic axis indicates the ability to represent information by the landscape. If two attractors coexist along the axis (bistability) and are mapped onto different fragments of information, e.g., two different goal positions, the characteristic axis is able to discriminate between them. On the other hand, if the dynamics is monostable, no information is represented on the characteristic axis (see Figure S1). Therefore, we hypothesize that the formation of cell assemblies in the PFC is updated depending on the task context and that the switching of information representation on the axes is produced with the reorganization of the attractors.

### Dynamical Models

We have proposed that the representational switching is achieved in the PFC by the abovementioned mechanism. In the initial stage of the goal-oriented action-planning task, the PFC network stays in the goal-representation mode and is ready to discriminate which task-relevant goal will be displayed. When the goal position is specified by a sensory input, PFC neurons maintain this information as persistent activity of the goal-representation mode in the neural network, and then, the state of the network is switched to the action-representation mode due to short-term plasticity to be explained below. Consequently, one of the action directions is selected by the convergence of the network state into one of the attractors in the action-representation mode. The selected action in the PFC network will be read out by downstream neurons, which may correspond to neurons in the motor cortex. Here we assumed that the read-out neurons are activated when the state of the PFC network has converged to the action representing attractor, namely when most of neurons in the action representing cell assembly are activated. The sequences of capturing sensory information, maintaining goal information, and transforming it into an action direction are executed as dynamical processes in the PFC network.

In the prefrontal network, neuronal responses are relatively diverse. Some neurons are involved in representing a specific goal or action during the entire task period, and others are involved in representing both of them and switching their representations during the task. Thus in general, the combination of functional cell assemblies may be more complicated (see Figure S2). However, in the present study, we have focused on essence of the observed phenomena and considered a minimal model. When one of the goals is displayed in the action-planning task of Figure 1A, the possible actions are actually limited to two directions. Therefore, for simplicity, we consider a PFC network that selects an action from the two possible actions cued by the displayed goal position. We implemented this mechanism to the dynamically reorganizable attractor network shown in Figure 2C. Each node in the figure indicates a population of neurons in the PFC (A to D), sensory neurons (G1 and G2), and read-out neurons (A1 and A2). Four neural populations in the PFC (A to D) are assumed to be mutually connected with three different types of excitatory synapses with or without short-term plasticity: namely, facilitation, depression, and constant synapses [22], [23] (see Methods for the detailed network structure). A given presynaptic neuron can form depression synapses on one neuron and facilitation synapses on another [22], [23]. The amplitude of the excitatory postsynaptic potential (EPSP) induced by a facilitation (depression) synapse increases (decreases) with successive presynaptic spikes, whereas constant synapses do not change the EPSP amplitudes. The excitatory neurons in the network are mutually connected and send excitatory output to a population of inhibitory interneurons through constant synapses. These interneurons send inhibitory synaptic outputs through constant synapses back to all excitatory neurons.

We assume that, in the initial resting state of the network when the synapses are still neither depressed nor facilitated, populations A and B as well as populations C and D form cell assemblies with relatively strong synaptic connections and encode two goal positions (see Figure 2D). These cell assemblies are mutually inhibiting via inhibitory interneurons, and thus the network should be bistable with the two active states of these cell assemblies [17], [29], as shown in the left of Figure 2D.

The neurons in the nodes that form such a cell assembly are assumed to be predominantly connected by synapses with short-term plasticity such that when one of these cell assemblies of A&B or C&D is selectively activated by the goal display, the cell assembly temporally holds the displayed goal position as working memory but subsequently loses its stability because of the dynamic modulation in synaptic efficacy. We further assume that when a cell assembly encoding a goal position becomes unstable, the synaptic modulation reconfigures active cells such that a cell assembly of A&D or B&C that encodes the action directions emerges in the network as a dominant cell assembly in turn, as shown in the right of Figure 2D [30]. Before and after the reconfiguration of cell assemblies, the representational modes of goals and actions are characterized by different patterns of bi-stability among cell assemblies that are partially overlapped across different modes. Therefore, the representational switching is not simply a change of cell assemblies but rather a higher-ordered reorganization of partially overlapped dominant cell assemblies based on multiple stability in the neural network. We examined plausibility of this dynamical mechanism with two types of computational models, namely, a spiking neural network model and its mean field model. Moreover, we evaluated several connectivity patterns by combinations of depression, facilitation, and constant synapses, and confirmed that the abovementioned reorganization of cell assemblies is robustly realized in these different connectivity patterns (see Methods).

### Representational Switching in Spiking Neural Network

First, we consider a spiking neural-network model in which each population of the excitatory neurons in Figure 2C is replaced with 200 noisy and leaky integrate-and-fire neurons. In each neuron, the dynamics of the membrane potential and the three different types of synapses are simulated. When a neuron receives many excitatory inputs and its membrane potential reaches a threshold value, the neuron generates a spike, and the synapses on the axon terminals of the neuron are activated. If the neuron generates a series of spikes, the efficacy of each synapse is modulated by the amount of the available synaptic resources (*x*) and the utilization parameter (*u*) that defines the fraction of resources used by each spike [23], [31]. The synaptic conductance induced by a synapse is determined by these two variables and a constant absolute value of the synaptic efficacy. The differences in the three types of synapses are based on the release probability of the neurotransmitter [23], [32], [33], and modeled with the different recovery-time constants of the available resources and the utilization parameter (see Methods for details).

**Figure 3:**
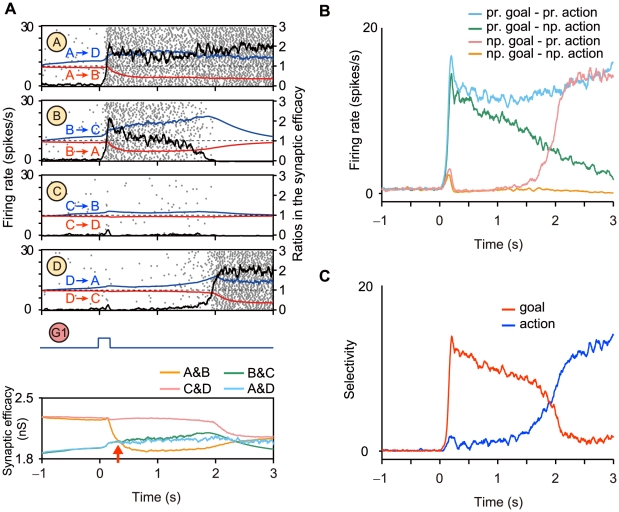
Simulated representational switching in a dynamically reorganizable attractor network model with spiking neurons. (**A**) Typical responses of the network consisting of all three types of synapses, namely facilitation, depression, and constant synapses (see Methods) when the sensory neural population G1 activates populations A and B in the prefrontal network. Black curves indicate the firing rates (the left coordinate). Blue (red) curves indicate the average facilitation (depression) ratios in the synaptic efficacy (the right coordinate). The ratios at the resting state are indicated by dashed lines. Each gray dots shows the timing of firing (50 of 200 neurons are displayed). The activation of a population of neurons induces facilitation and depression in these synapses and causes a state transition in the middle of the delay period. The bottom panel shows a transition of the average synaptic efficacy on each cell assembly. The red arrow indicates timing of the transition. (**B**, **C**) These figures are in the same format as Figures 1B and 1C. (**B**) Responses of a population of excitatory neurons for preferred (non-preferred) goals and actions. (**C**) Selectivity measures that show representational switching from the goal-representation mode to the action-representation mode.

Figure 3A shows a typical response of the network consisting of all the three types of synapses, namely, facilitation, depression, and constant synapses when the goal position G1 was presented as a sensory input at the beginning of the goal-display period. The network shows a state transition from one active state of a cell assembly (A and B) to another (A and D). After the state transition, the activation of the cell assembly (A and D) can be read out, which would activate motor neurons that encode the action direction A1. In this network, goal and action encoding cell assemblies are predominantly connected by depression and facilitation synapses, respectively. During the goal-display period, the synaptic connections between A and B in the cell assembly activated by the goal display (see Figure 2C) were gradually depressed (see the red curves in Figure 3A), and the connections from A to D and from B to C in the cell assemblies that represent actions were facilitated as shown in blue curves in Figure 3A. The time-varying synaptic efficacy in a cell assembly is quantified with the average peak synaptic conductance in a given cell assembly (see Methods). Dominant cell assemblies that have greater synaptic efficacy were switched from the goal-cell assembly A&B to action cell assemblies A&D and B&C (see the red arrow in Figure 3A bottom), and the active state that represents the goal position (the cell assembly with A and B in Figure 3A) is disbanded and another active state that represents the action (the cell assembly with A and D in Figure 3A) is formed. Because the connections among neurons in a cell assembly representing the action are facilitated, this active state is stable. Note that this stable action-representation state can be reset to the initial goal-representation state by decreasing the applied activation input.

Figures 3B and 3C show that the model qualitatively captures the main features of the experimentally demonstrated responses and the representational switching as shown in Figures 1B and 1C. These results were obtained from average firing rates of populations of excitatory neurons in 40 simulation trials, including four possible patterns of state transitions consisting of all the combinations of two goals and two actions. Some disagreement between Figure 1B and 3B may be due to a diversity of the response properties of neurons in the PFC (see also references [2], [4]), whereas we assumed uniform parameter values for model neurons in the present model. This simulation was based on a network consisting of all three types of synapses. We confirmed that these simulation results that show the representational switching can be also obtained from a network consisting of only depression and constant synapses or of only facilitation and constant synapses. Even in these networks consisting of a single type of short-term plasticity, the switching of dominant cell assemblies from a goal to an action is also observed (see Figure S3).

**Figure 4 pcbi-1002266-g004:**
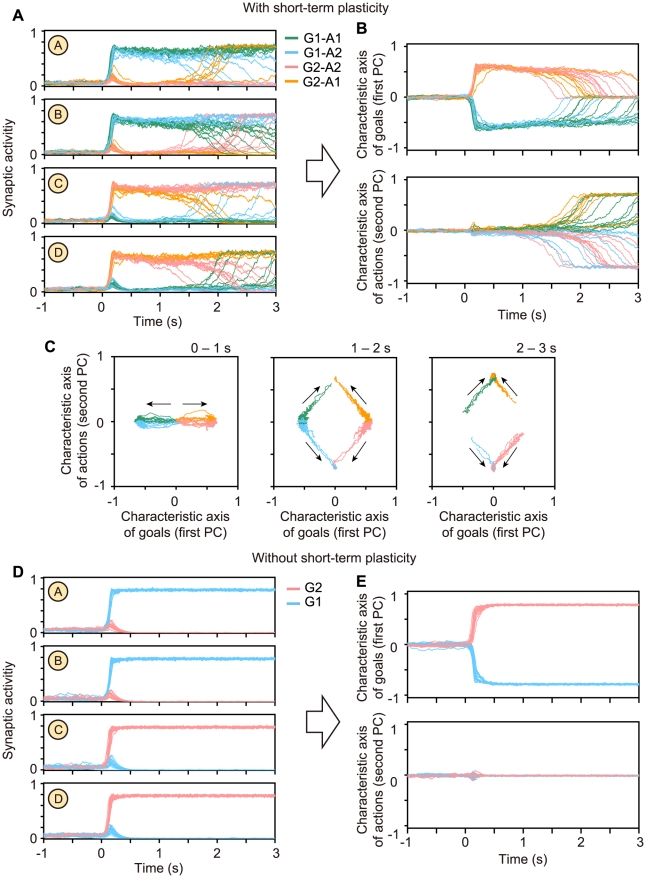
Properties of the dynamically reorganizable attractor network model with spiking neurons. (A) The responses of 40 simulation trials in four populations of neurons and (**B**) those in the first and second principal components (PCs). The color code is based on the goal positions (G1 and G2) and the action directions (A1 and A2). Goals and actions are separated in the first and second PCs, respectively. (**C**) A phase-plane view of (**B**). Initially, the network was at the resting state, which is the origin of the phase plane. The state then moved to one of the active states present on the first PC axis, which represents a goal. The state of the network stayed on the first PC axis for approximately 1 s (left). Then, the state moved to one of the active states present on the second PC axis (center). Finally, the state converged to the active state, which represent an action (right). (**D, E**) The responses without short-term synaptic plasticity in the same format as (**A, B**). If synaptic connections were fixed, the network did not show any transition with representational switching.

Next, we used a dimension-reduction formulation based on principal component analysis, which resulted in a multivariate trajectory of the population activity in the model transformed into its first and second principal components (PCs) (see Text S1). The trajectories of four different patterns of state transitions were separated in PCs (Figures 4A–4C). The trajectories were distributed along the first (second) PC axis before (after) the representational switching (Figures 4B and 4C). This result suggests that the first and second PCs can be regarded as the characteristic axis of goal positions and that of action directions, respectively.

How does the short-term synaptic plasticity contribute to the representational switching? We confirmed that the representational switching does not occur if the synaptic efficacy is fixed in the network. In the absence of any short-term plasticity, a sensory input triggered the activation of a cell assembly that encodes a goal position; however, the network did not show a state transition to another state that encodes an action direction (Figures 4D and 4E).

**Figure 5 pcbi-1002266-g005:**
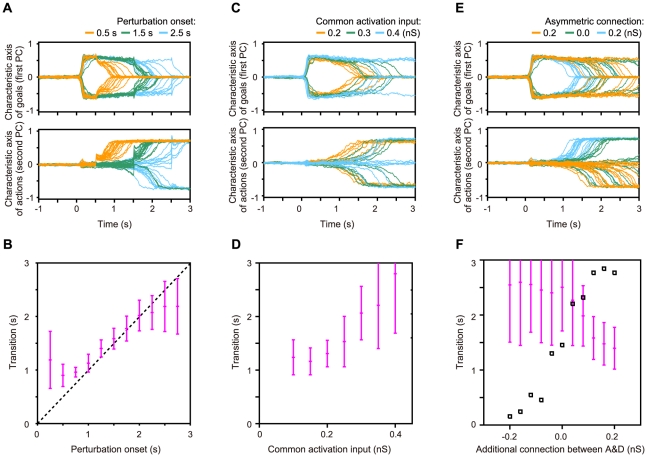
Changes of transition time from goal representation to action representation. (**A, B**) The responses to perturbation inputs. The onset of the perturbation input expedites the transition. (**B**) The transition time as a function of the onset time of the perturbation input. (**C, D**) The responses with different values of the common activation inputs. Increase in the common activation inputs leads to a delay in the transition time. (**E, F**) The responses with asymmetric connections in action-representing cell assemblies. Greater connections within one of the action-representing cell assemblies, which is composes of nodes A and D, advance the transition time and increases the tendency of transitions to this cell assembly with nodes A and D. In (**F**), the square indicates the ratio of transitions to this cell assembly. Figures 5 A, C, and E show 20 simulation trials for three typical parameters on the first and second principal components.

To examine the effect of the short-term plasticity on the network stability as well as a possibility to control the timing of the switching, we applied a small perturbation input to the network. Then, the state transition occurred earlier due to the perturbation input (Figures 5A and 5B). The interval between the perturbation and the state transition was large immediately after the onset of the goal display. In contrast, the interval was small when the perturbation onset was close to the proper timing of the transition. These responses indicate that after the activation of the goal-encoding cell assembly, the network gradually lost stability and became increasingly susceptible to fluctuations in the neural activity. In real experiments, depending on the task, the delay period can be varied and animals can follow this change, suggesting flexible modification of the transition time. This modification can be realized by the abovementioned perturbation to the PFC network. Unexpected sudden appearance of the “Go” signal, for example, may affect the PFC network as a perturbation.

In addition, the transition time can be also modulated by the common activation inputs. Greater activation inputs induced delayed transitions (Figures 5C and 5D) because the inputs may cause more stabilization in an already activated cell assembly.

In the above results, we considered a case in which the connectivity in two cell assemblies encoding action directions are symmetric, implying that the two possible action directions were randomly determined with equal probability although a specific goal position generally has the tendency to lead to a specific action direction. We confirmed that such a general correlated tendency of the state transition can be implemented with asymmetric connectivity. If one of two action-representing cell assemblies has greater mutual connections than the other, the tendency of selection of this corresponding action is increased, and the transition time is reduced with increasing the asymmetric connectivity (Figures 5E and 5F).

### Stability Analysis Based on a Mean Field Model

The results above are based on a network model composed of spiking neurons, the dynamics of which is defined by thousands of variables. Thus, it is difficult to analyze the underlying population dynamics of this intricate spiking neural network. This difficulty can be alleviated with a mean field approach. Therefore, we considered the means of synaptic activity (*s*) and the variables that define the short-term plasticity: namely, the available synaptic resources (*x*) and the utilization parameter (*u*). In each neural population in the model, the mean variables were dependent on a population-averaged firing rate that is given as a function of the conductance induced on the neural population (see Methods for details).

**Figure 6:**
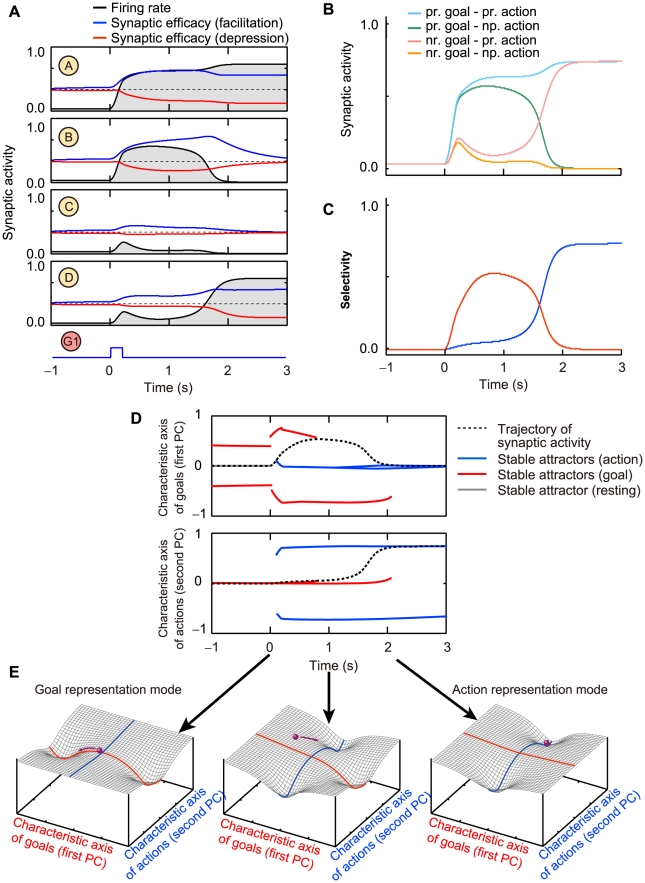
The responses of the mean field model and stability analysis. (**A**) The time courses of synaptic activity in the populations of the excitatory neurons, A to D, that are in the same format as in **Figure 3A**. (**B, C**) The properties of the synaptic activity and selectivity that are in the same format as in **Figures 1B and C**. (**D**) The temporal evolution of stable attractors in the coordinates of the first and second principal components (PCs), indicating the characteristic axes of goals and actions, respectively. (**E**) Schematic attractor landscapes with respect to the first and second PC axes. Positions of the attractors are indicated by the valleys of the surfaces on the basis of the analysis in (**D**). The purple balls show the state of the network and move following the attractor landscape. The attractors that contribute to the representation of goals (red curves) are situated on the first PC axis, while those that contribute to the representation of actions (blue curves) are situated on the second PC axis.

The responses in the mean field model are qualitatively similar to those in the spiking neural network (Figures 6A–6C); its trajectories are smoothened owing to the absence of noise.

The underlying mechanism of the representational switching and the subsequent change of the neural activity might become clear by considering the stability that can explain the formation of attractors. The timescales of the dynamics of the membrane potential, the spike generation, and the synaptic activity are relatively faster than those of the synaptic modulation with the short-term plasticity. The time constants of the synaptic activity are less than 100 ms. In contrast, the recovery-time constants of the available resources and the utilization factors in the synaptic modulation are approximately 500–1000 ms [22], [23]. Therefore, the fast dynamics is dominated by the slow dynamics of the synaptic modulation variables that work as bifurcation parameters, whereas the slow variables are also influenced by the fast dynamics. We analyzed how the stability of the fast dynamics is modulated by the slow dynamics. A dissipative dynamical system like neural network models can be generally characterized by the concept of attractors. We thus applied an equilibrium-finding algorithm and stability analysis to a dynamical system of the synaptic activity that is dominated by the synaptic modulation (see Methods for details). On the coordinates of the first and second PCs, we traced how the stability of the attractors is modified by the synaptic modulation (Figures 6D and 6E).

Stable attractors were formed depending on the synaptic modulation, and the state of the synaptic activity follows the formation of the attractors. The system initially has three stable attractors: the resting state located on the origin of the first and second PC coordinates, and two attractors encoding the goal positions (the red curves in Figure 6D). These two goal-encoding attractors were situated on the first PC axis, and correspond to the active state of populations A and B and that of populations C and D, respectively. The resting state was destabilized by the sensory input that reflects a goal position, and the state of the network moves to the attractor that represents the displayed goal position. When the state of the network approaches the goal-representation attractor, the goal-representation attractor starts to become unstable because of the synaptic modulation. Then, two stable attractors appears on the second PC axis, which represent actions (the blue curves in Figure 6D). The state of the synaptic activity converges to an action-representation attractor and the attractor was maintained. Thus, the information representation of the network is ascribed to the dynamical formation of attractors, which can be updated by a sequence of stabilization and destabilization of the attractors due to the synaptic modulation.

## Discussion

The PFC exhibits highly flexible representation of information. Even a single neuron seems to encode multiple sets of information and switch the representation dynamically and internally, depending on the task context. We hypothesized that this representational switching is based on the modulations of synaptic connectivity and can be described as a bifurcation process of attractors in the dynamical system that describes the nonlinear dynamics of neural activity in the neural network model. In this study, we proposed a dynamically reorganizable attractor network model with short-term synaptic plasticity as a minimal model that explains representational switching in the goal-oriented action-planning task. Our results demonstrated that cell assemblies encode the required information by forming multiple attractors and that these cell assemblies are reorganized by synaptic modulation with short-term plasticity such that the network is able to encode other sets of information. The properties, obtained by mathematical modeling with the spiking neural network and its mean field version, are consistent with those of neural activity in the monkey PFC during the goal-oriented action-planning task.

### Underlying Mechanism of Representational Switching

Experimentally observed neural activity in the PFC implies that fragments of information about goals and actions are represented as sustained activity of PFC neurons and that possible patterns of neural activity are limited depending on goal- and action- representation mode (see Figure 1E). Representations with cell assemblies dynamically vary with an internal mechanism of the PFC network.

Conventional attractor frameworks can explain the representation of information with sustained neural activity [12], [14], [34]. Namely, an active state of a cell assembly constitutes an attractor and represents a fragment of information. In most such conventional views, the attractors structure is assumed to be static or time-invariant. Although an active state of whole the network shows a transition from one cell assembly to another one by external stimuli or noise produced by neural spiking (see for example [18]), the attractors structure and the encoded information is invariant.

However, in the representational switching observed in the PFC, a formation of cell assemblies and the encoded information in these cell assemblies are varying spontaneously depending on the task context without external signals. In the present model, the formation of cell assemblies is dynamically reorganized with synaptic modulation. In an initial stage of the task context, the information required by the task context is mapped onto some cell assemblies, the activity of which is triggered by a specific population of sensory neurons. Then, the reorganization of attractors with other cell assemblies is internally induced by the synaptic modulation. Finally, the state of the network move to the newly formed cell assemblies through bifurcations and its activity is read-out by another population of neurons, e.g. motor neurons.

This dynamical reorganization of functional cell assemblies can be interpreted as reorganization of “synapsemble” [19], or an assembly of synapses. The activation of the cell assembly induces depression of synapses in the cell assembly, and simultaneously induces facilitation of synapses among other neurons that form other cell assemblies. In this process, dominant cell assemblies switch due to change of synaptic efficacy (see the red arrow in Figure 3A bottom); this switching can be also achieved in networks consisting of only depression and constant synapses or of only facilitation and constant synapses as shown in Figure S3. Accordingly, the initially required information is transformed to another information by forming the subsequent representational state, and this information is consecutively read out by another population of downstream neurons.

These multiple representations of information are characterized by the dynamical attractor landscapes shown in Figure 6E. The synaptic modulation destabilizes initially required attractors and then stabilizes other attractors required at the subsequent stages of the task context. The positions of the attractors are situated, first on the initial representation axis and then on the subsequent representation axis. This functional degeneration of the dimension and switching of the dynamics on the characteristic axes are the essence of the representational switching. In contrast with the conventional attractor framework where transitions among static attractors are triggered by external stimuli or noise [18], we proposed a new dynamical viewpoint that short-term synaptic modulation causes changes in attractors structure of the state space and that the state transition occurs through bifurcations on the basis of the dynamically changing attractor structure. As a future study, this mechanism should be verified with further experimental data. Generally speaking, nonlinear dynamical systems exhibit characteristic behavior just before the state transition by bifurcations, e.g. increases in fluctuation and correlation [35]. There are possibility to evaluate this characteristic behavior with further data collection and analyses.

In the present model, the representational switching of the reorganization of cell assemblies relies on the inhomogeneous connectivity of the depression and facilitation synapses (see Methods for details about this connectivity). How is this inhomogeneous connectivity acquired or learned from experience? This can be achieved, for example, by Hebbian learning [18], [36] as well as reinforcement-based learning [37], [38] in which synaptic connections that contribute to achievement of rewardable behavior are selectively strengthened. In the initial stage of the learning, facilitation and depression synapses may constitute a random network with a homogeneous distribution (see Figure S4). Despite this homogeneous connectivity, the synaptic connections between pairs of neurons vary in type of synaptic connection (i.e., depression or facilitation) and in the intensity of synaptic efficacy. The neural network shows diverse responses for each trial even under the same experimental condition because of the randomness of the timing of spike generation. In the process of learning, when the activities of a pair of neurons correlate with each other and contribute to achievement of rewardable behavior, the synaptic connections between this neuronal pair will be enhanced. Correlated activity among neurons enhances the synaptic connections between these neurons through Hebbian learning, and further, reward-based synaptic modulation may lead to more enhancements of synaptic connections through reinforcement learning. For example (see Figure S4B), the goal encoding sensory neurons G1 activate some neurons in the PFC (including candidates of neurons in populations A and B in Figure 3). These neurons have correlated activity due to simultaneous activation, and thus the synaptic connections between them are enhanced by Hebbian learning. These neurons may have relatively strong connection to other PFC neurons (including candidates of neurons in population C or D), which may be easily excitable due to these strong connections. Further, some of these PFC neurons (e.g., neurons in candidates of the population D) may have reciprocal synaptic connections to the PFC neurons that are directly activated by the sensory input (i.e., a neuron in candidate of the population A). Moreover, some of these PFC neurons have connections to the read-out neurons that represent actions. Activity of these neurons initiated by the sensory neurons G1 can be correlated, and thus the synaptic connections between them tend to be enhanced by Hebbian learning. Furthermore, if the activity of the neurons contributes to obtaining a reward, namely, if facilitation synapses contribute to representation of action with stable activation of cell assembly and if depression synapses contribute representation of goal with temporal activation of cell assembly, reward-based learning will effectively reinforce synaptic connections that have contributed to the rewardable behavior, namely those connections that have been activated at the moment of or just before the reward acquisition [37], [38], [39], [40]. Strengthened synapses with these learning rules may contribute to the obtainment of more reward on subsequent trials. Such learning rules in consecutive trials may result in the formation of functional subnetworks with inhomogeneous connectivity. Indeed, the abovementioned reciprocity with specific types of synapses is observed in the PFC [22]. Moreover, the timing of the representational switching is sensitive to the strength of synaptic connections (e.g., Figure 5E). Thus, the appropriate timing of the transition is also adjustable with these learning rules. It should be noted that neural network models can reproduce transitions from a retrospective to a prospective activity during a delay period through Hebbian learning and fluctuation [18]. A similar mechanism may also work to realize the representational switching, for example, from the cell assembly of populations A and B to that of A and D. In this sense, although our model provides a new mechanism for the representational switching based on a dynamical reorganization of the attractor landscape, which is different from the well-known mechanism of transitions among attractors in the static attractor landscape, detailed analysis of learning and neural dynamics on representational switching in the PFC still remains to be explored.

### Roles of Representational Switching in Prefrontal Lobe

The self-organized transition demonstrated by our dynamical model can help to understand recent studies on changes in the information representation coded by cell activity in the cerebral cortex [41], [42], [43], including the frontal cortex [2], [4], [29], [44]. The transition on the characteristic axes, which is an important aspect of our model, seems to be particularly consistent with the aspect of executive control of behavior, which is believed to be attributed to the PFC [45], [46], [47]. Such a transition can serve as a fundamental mechanism of the executive function that requires qualitative transformation between different categories of information. For example, the transition on the characteristic axes could correspond to set-shifting in the Wisconsin Card Sorting Test (WCST). It should be noted that representational switching in WCST is also explained by a recurrent neural network model with neurons randomly connected both to the recurrent network and sensory inputs [48].

The prefrontal executive function might be a basis for our creativity [49]. Creativity almost always involves emergence of a novel axis or dimension in cognition and behavior, which is impaired by frontal-lobe damage [50], [51]. Such aspects behind creativity can be the transition to a new representational axis demonstrated in our dynamical model, which may serve as a fundamental neuronal mechanism.

The PFC is thought to be on the top of the functional hierarchy of voluntary actions and is making decisions about action generation with their internal process rather than with an external stimulus or with a signal from other parts of the brain. The representational switching occurs without external cues as shown in the present task (see Figure 1). If the representational switching is assumed to be triggered by an attentional signal from other part of the brain, it contradicts the fact that the PFC is on the top of the functional hierarchy and requires that the decision is performed by other parts of the brain.

The interval between occurrence of the state transition and the Go signal onset (∼1 s) is longer than time constants of the synaptic activity and modulation. The timing of the state transition is determined by the stability of the whole network dynamics rather than by the dynamics of individual neuron or synapse. Generally speaking, a nonlinear dynamical system becomes slowing down and increases in sensitivity to small fluctuation just before the state transition [35]. In the present model, the slowing down of network dynamics and the fluctuation in the neural activity lead to large deviation in the timing of the state transition and results in the correlation tendency between the mean and the deviation in timing of the state transition as shown in Figure 5. Further, state transitions that occur with specific time delays from a cue onset may contribute to a representation of interval timing. The property of the timing of the state transition shown in the present model has an agreement with the scalar property [52] in which the mean and the standard deviation of the response time of an animal covary in an interval-timing task.

Immediately before state transitions, nonlinear dynamical systems generally become sensitive to small perturbations and can easily trigger state transitions [35], [53]. This idea has been applied to modeling of dynamical aspects of brain functioning [54], [55] and is also demonstrated in our model (Figure 5A and 5B). However, the “trigger” should not be mere noise, considering the nature of creativity or thought. Creativeness, or devising a new viewpoint and dimension, involves finding coherent relationship between internal and external information represented in the mind [56], which may emerge as the transient synchrony of neural activity [57], [58]. This idea was also supported by our previous study in which transient neuronal synchrony was enhanced around the representational switching of behavioral goals [2], which is consistent with the results provided by our model (compare Figure 5B with Figure 5C of Sakamoto et al., 2008). It is an important future problem to explore a comprehensive view of neuronal dynamics, including transitions and synchrony.

### Further Future Studies

In the present study, we have focused on the short-term synaptic plasticity as a component of the PFC network. However, the short-term plasticity is only one of many time-dependent properties that influence synaptic connectivity and have the potential to explain the representational switching. Other influential modulations in synaptic connections can be caused by monoaminergic neurotransmitters such as dopamine [34] and acetylcholine [59] as well as spike-timing-dependent plasticity [60]. Possible mechanisms of representational switching that include these components should be investigated in future.

Here we have presented a minimal model to explain the representational switching between only two sets of binary information. In our hypothesis, the coexistence of multiple representations in a single network relies on the dynamical formation of cell assemblies. Thus, in principle, a single neural entity is capable of becoming more flexible and encoding more than two sets of information (see Figure S2).

The present model is limited on the representation of sets of discrete information and an abstract aspect of sensory and motor information. Besides the discrete information, the PFC represents parametric information, e.g. intensity of a task related sensory stimulus or a coordinate of an arm movement. Transformation between such kinds of parametric information may contribute, for example, to a visio-motor coordinate transformation, which may be processed mainly on lower areas of the functional hierarchy of the brain, e.g. motor cortex or cerebellum. Representations of such parametric information may be achieved not only by attractors but also by trajectories of transitions among attractors [61]. The coordination between different areas of the functional hierarchy and between different kinds of discrete and parametric information remain to be further investigated.

Moreover, the reorganization of functional cell assemblies can sequentially occur among more than two modes of representation. In our daily lives, we are required to handle many different categories of information as well as the step-by-step switching between them. Thus, our hypothesis should be further evaluated in such usual situations.

## Methods

### Ethics Statement

The physiological experiments were performed on animals cared for in accordance with the Guiding Principles for the Care and Use of Laboratory Animals of the National Institutes of Health, and the Guidelines for Animal Care and Use of Tohoku University.

In the present study, we used two approaches for modeling neural networks in PFC, which are depicted in Figure 2C. The first approach is modeling with spiking neurons that simulate the generation of spikes, the synaptic activity, and the synaptic modulation with short-term plasticity, including their stochastic properties. The other approach is a mean field model that simulates the population averages of the variables and allows us to analyze a skeleton of the underlying population dynamics.

### Spiking Neural Network

To model the spiking network of PFC, we used noisy and leaky integrate-and-fire neurons with dynamic synapses that undergo synaptic plasticity. Each neural population from A to D and IN in Figure 2C consisted of 200 integrate-and-fire neurons; in total, 1000 neurons were used. The membrane potential of each neuron in each population of neurons 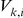, (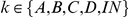, 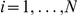 with *N* = 200) varies according to the following equation [15]:
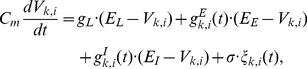
(1)where 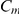 is the membrane capacitance and 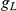 is the conductance that induces leakage currents. 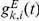 and 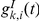 are the conductances on excitatory and inhibitory synapses, respectively, induced by other presynaptic neurons and external inputs. 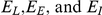 represent the corresponding reversal potentials. When 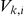 reaches the threshold value of the membrane potential 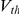, the neurons generate an action potential or a spike, and the membrane potential is reset to the resting potential 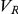 and maintained at this potential level during the absolute refractory period 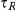. 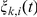 represents Gaussian white noise that is applied for each neuron independently with mean 0 and standard deviation 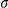.

When a neuron generates a spike, synapses on the axon terminals of the neuron are activated, and generate synaptic currents on the postsynaptic membranes. This postsynaptic current is modeled with a variable that represents the synaptic activity, or the ratio of open receptor channels in the postsynaptic terminal. The dynamics of the synaptic activity depends on excitatory or inhibitory synapses and the types of properties of short-term plasticity. In the present model, we used three types of excitatory synapses, namely, facilitation, depression, and constant synapses [22], [23], the synaptic activity values of which are denoted by 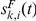, 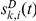, and 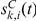, respectively, where *k* and *i* are indices of a neural population and a neuron to which the synapses belong, respectively.

The conductance induced by a synapse is given as the product of the weight of synaptic connection and the synaptic activity. We assumed that the absolute magnitude of the conductance and its synaptic type of short-term plasticity are common in all the synaptic connections from one population to another population, and that each neuron receives a constant bias input and a time-varying external input. Thus, the conductance is defined by the following equation:
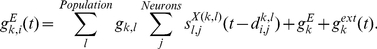
(2)In the first term on the right-hand side of equation (2), 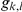 denotes the weight of synaptic connection from population *l* to population *k*. 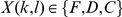 specifies the type of excitatory synapses connected from population *l* to *k*, where *F*, *D*, and *C* indicate facilitation, depression, and constant synapses, respectively. 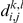 is the transmission delay from the *j*th neuron in population *l* to the *i*th neuron in population *k*, which is uniformly distributed from 1 ms to 5 ms. In equation (2), the first and second summations run over connected presynaptic neural populations and connected presynaptic neurons in these neural populations, respectively. The second term 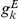 denotes the constant bias conductance. The third term 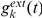 is the time-dependent external input that describes both activation and sensory inputs. Activation inputs are commonly applied to the neural populations A to D in the form of a piece-wise linear function after the onset of the goal display during the task period. After the onset, the input magnitude linearly increases from 0 to 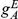 until time 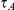, and remains at 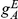 after 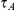. The activation input results in the active state of a goal-representing cell assembly by destabilizing the resting state. The sensory input is applied to a cell assembly representing one of the goals (a pair of A and B or a pair of C and D) from the onset of the goal display as a rectangular pulse with amplitude 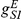 and width 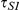.

The synaptic activity of an excitatory synapse is modulated by short-term plasticity and modeled as follows. Each presynaptic neuron triggers three types of synaptic activity. The synaptic activity 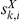 is set to a peak value 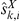 by a presynaptic spike, and exponentially decreases to zero with a time constant 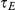 [29] as follows:
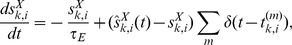
(3)where 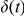 is the Dirac delta function, and 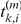 denotes the time of occurrence of the *m*th spike in neuron *i* in population *k*. In the case of constant synapses, the peak value of synaptic activity is fixed at unity. In contrast, the peak value of synaptic activity with either facilitation or depression plasticity is time-dependent, and is given by the product of the utilization factor 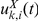 that defines the fraction of resources used by each spike and the amount of available resources 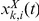 as follows [23], [31]:
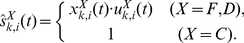
(4)Variables 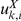 and 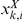 vary according to the following equations:
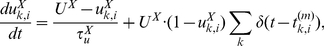
(5)

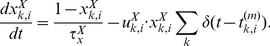
(6)Equations (5) and (6) describe dynamics of the facilitation (*X* = *F*) and depression (*X* = *D*) synapses. The difference in the types of the short-term plasticity is determined by the resting state of the utilization factor 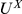 and the recovery-time constants from depression and facilitation, 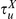 and 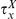, respectively.

Regarding inhibitory synapses, all synaptic connections are derived from the population of interneurons, the synaptic activity of which is denoted by 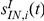, and the conductance induced by the inhibitory neurons is given by
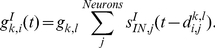
(7)


We assume that all inhibitory synapses are constant synapses for simplicity, i.e., the peak value of synaptic activity is fixed at unity as follows:
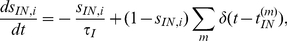
(8)where 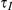 is the synaptic time constant.

The synaptic efficacy in a cell assembly is defined as the average of the peak conductance of excitatory synapses on neurons in the cell assembly. The peak synaptic conductance in each neuron is given by
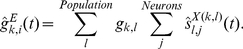
(9)


### Network Structure

Figure 2C shows the overall network structure. The populations of neurons in the nodes of Figure 2C, which represent PFC neurons (A to D and IN), are simulated with the spiking neuron model as explained above. The PFC neurons are driven by a sensory input that represents the goal positions (G1 and G2). The activity of the PFC neurons is read out by the populations of the neurons (A1 and A2). Although sensory and motor neurons are depicted as neural populations (G1, G2, A1, and A2), their activity was not explicitly simulated; the activity of G1 and G2 is introduced by bias inputs for the PFC neurons and the activity of A1 (A2) is given by summed activity of A and D (B and C). Each population of neurons in the PFC network consists of 200 integrate-and-fire neurons. The neurons are sparsely connected within and across the nodes. Suppose that the connectivity ratio *c* is 0.2, and each neuron receives randomly selected *cN* presynaptic connections from each presynaptic neural population. The weight of a synaptic connection from population *l* to *k* is defined as follows: 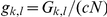, where 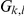 is the summed weight of the connections from population *l* to *k*.

### Mean Field Model

Based on the integrate-and-fire neuron model, we constructed a mean field model that simulates the mean activity of a neural population, and allows us to define the overall dynamics of many populations of neurons and analyze changes in the stability. In the present model, the mean firing rates of excitatory and inhibitory neurons are denoted by 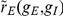 and 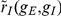, which are the functions of excitatory and inhibitory input conductance 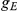 and 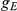, respectively. The input conductance can be approximated as linear combinations of the excitatory and inhibitory conductances, namely, the firing-rate response function can be approximated with the coefficients 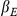 and 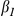 as follows [29]:
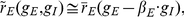
(10)

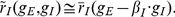
(11)


In the present study, the approximated form of the firing-rate response function is given as the following Naka–Rushton formula [62], which is generally used to fit intensity-response curves:
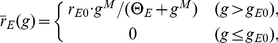
(12)

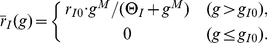
(13)


The parameter values in equation (12) and (13) were determined to respectively fit the responses in the abovementioned excitatory and inhibitory spiking neurons. Using this formulation, the mean firing rate of population k in the present network is given by
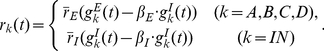
(14)


Similarly to equation (2), the excitatory conductance induced in the *k*th population of neurons is
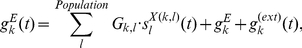
(15)where 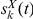 represents the mean synaptic activity that changes depending on the type of synapses. The mean activity of constant synapses was modeled as follows. When the neurons in a population fire asynchronously, the mean synaptic activity in the population should be almost stationary. When the mean synaptic activity changes because of changes in the firing rate, the synaptic activity will converge to the stationary value with a certain time constant. If each neuron fires with firing rate *r* and if the peak of the synaptic activity is unity, the temporal average of the synaptic activity is 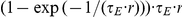. Here we assume that the mean synaptic activity converges to this value with the time constant 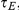, i.e., the synaptic activity obeys the following equation:
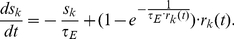
(16)


In the case of synapses that undergo modulation with short-term plasticity, 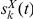 can be denoted as a combination of the mean synaptic activity of constant synapses 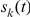 and a term of the synaptic modulation by the short-term plasticity as follows:
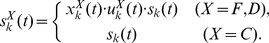
(17)


Similarly to equations (5) and (6), the means of the utilization factor 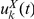 and the available resources 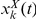 change according to the following equations [31]:
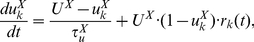
(18)

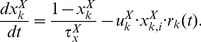
(19)


The inhibitory conductance and its synaptic activity are analogous to equations (7) and (8) as follows:
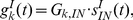
(20)

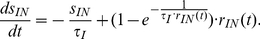
(21)


The network structure of the meanfield model is the same as that of the spiking neuron model. The absolute strengths of all connections are denoted as the summed weight of connections, as shown in equation (15).

In the mean field model, the dynamics of the synaptic activity 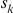 was defined by variables that indicate the synaptic modulation 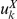 and 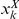. We traced the changes in the dynamical system for 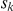 by identifying stable attractors. In each step of the numerical integration of the model, an equilibrium-finding algorithm (the Newton–Raphson method) and eigenvalue analysis were applied to identify the stability of attractors [63].

### Model Parameters

The differential equations were simulated by the Runge–Kutta method with the time step 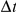 = 0.1 ms.

The following parameter values were used for the spiking neuron [15]. For both excitatory and inhibitory neurons, 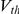 = −52 mV, 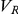 = −60 mV, 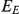 = −5 mV, and 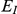 = −75 mV. For excitatory neurons, 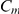 = 0.5 nF, 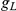 = 25 nS, 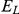 = −70 mV, and 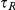 = 2 ms. For inhibitory neurons, 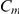 = 0.2 nF, 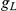 = 20 nS, 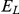 = −65 mV, and 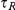 = 1 ms [15]. The stochastic term 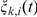 in equation (1) was simulated by adding a random variable following the normal distribution with mean 0 and variance 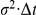 at each integration time step. We set 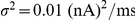.

For excitatory synapses, we assumed a long time constant 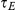 = 100 ms for N-methyl-D-aspartate (NMDA) synapses, which may be important to maintain the active state in the PFC network [14], [15] because this long time constant smoothens the destabilizing effect due to random spike activity. Although the dynamics of NMDA synapses is characterized by the long time constant and the membrane-voltage dependency, we used only the long time-constant aspect of NMDA synapses for simplicity. For inhibitory synapses, we set 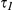 = 20 ms.

The magnitude of the constant bias input was set such that the neuron stays just below the firing threshold or exhibits very low-frequency firing: for excitatory neurons, 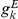 = 8.35 nS, and for inhibitory neurons, 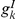 = 4 nS. For external inputs, the parameters of the activation inputs are set to 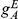 = 0.35 nS and 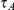 = 200 ms, and the amplitude and width of sensory inputs are 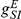 = 0.2 nS and 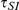 = 200 ms, respectively. For the short-term synaptic plasticity, we set 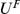 = 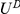 = 0.2, 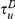 = 20 ms, 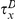 = 600 ms, 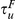 = 600 ms, and 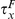 = 100 ms.

We constructed the following three types of network structures. The first consists of all the three types of synapses, and its simulation results are shown in Figures 3–6. The second and third types of networks consist of only depression and constant synapses, and only facilitation and constant synapses, respectively; the results with these networks are shown in Figure S3.

For the first type of networks, the summed conductance and types of synapses are as follows. For connections among goal-representing cell assemblies, 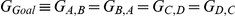 = 3.2 nS, 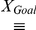 *X(A,B)* = *X(B,A)* = *X(C,D)* = *X(D,C)* = *D*. For connections among action-representing cell assemblies, 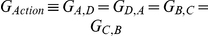 = 1.55 nS, 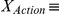 *X(A,D) = X(D,A) = X(B,C) = X(C,B)* = *F*. For self-recurrent connections, 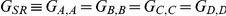 = 1.7 nS, 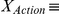 *X(A,A)* = *X(B,B)* = *X(C,C)* = *X(D,D)* = *C*. For connections from excitatory neurons to inhibitory interneurons, 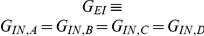 = 0.7 nS, 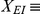 *X(IN,A)* = *X(IN,B)* = *X(IN,C)* = *X(IN,D)* = *C*. For connections from interneurons to excitatory neurons, 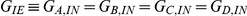 = 5 nS, 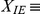 *X(A,IN)* = *X(B,IN)* = *X(C,IN)* = *X(D,IN)* = *C*.

For the second type of networks in which the short-term plasticity is driven only by the depression synapses, the summed conductance and types of synapses are as follows: 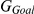 = 3 nS, 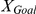 = *D*, 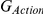 = 0.5 nS, 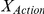 = *C*, 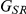 = 1.8 nS, 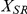 = *C*. 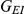 = 0.7 nS, 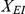 = *C*. 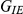 = 5.5 nS, 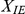 = *C*.

For the third type of networks in which the short-term plasticity is driven only by the facilitation synapses, the summed conductance and types of synapses are as follows: 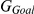 = 0.8 nS, 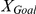 = *C*, 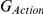 = 3.1 nS, 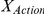 = *F*, 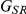 = 1.9 nS, 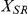 = *C*. 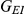 = 0.7 nS, 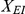 = *C*. 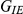 = 7.5 nS, 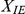 = *C*.

This model exhibits the representational switching in a wide parameter range. Although the timing of switching is sensitive to the strength of the synaptic connections and the parameters in the dynamics of the short-term plasticity, the timing was easily adjustable in an experimentally plausible range as shown in Figure 5.

For the mean field model, the parameter values in the firing-rate response curve were specified to fit responses in the population of the integrate-and-fire neurons. We set *M* = 2 and obtained, for excitatory neurons, 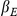 = 0.4611, 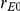 = 0.2561, 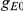 = 8.560, and 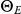 = 12.81, and for inhibitory neurons, 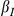 = 0.4611, 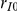 = 0.2561, 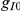 = 8.560, and 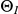 = 12.81. Note that the units are in kHz for the firing rates and in nS for the conductances.

The parameter values of the dynamic synapses and the connection strength are the same as those of spiking neurons, except for 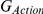 = 1.9 nS, because the spiking neural network shows an earlier state transition compared with the mean field model because of fluctuations in neural activity. Thus, we adjusted the connection strength such that the mean field model shows a state transition in a similar time range. If the model is completely symmetrical in its connectivity, the state of the network remains on a saddle point and does not show any state transition. Therefore, we applied very small perturbation inputs that induced state transitions at the beginning of the goal-display period. The perturbation input is applied to a cell assembly (a pair of A and D or a pair of B and C) representing one of the actions as a rectangular pulse with the amplitude of 0.01 nS and the width of 200 ms.

### Measure of Selectivity

The selectivity is determined using the firing rate, the goal position, and the action direction by the following equation: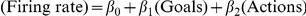, where 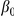 is an intercept, and the coefficients 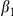 and 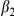 indicate the goal and action selectivity, respectively. The regressors “Goals” and “Actions” indicated in parentheses are dummy variables [27] that represent preference or non-preference of the neuron for goals and actions. For example, in the case of the present model, there are two goal positions and two action directions. These discrete variables can be represented by dummy variables 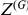 and 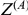 as follows:
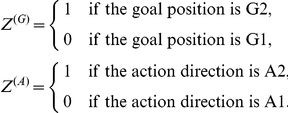



The regression model with these dummy variables is given by 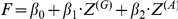, where *F* is the firing rate, and the goal and action selectivity 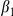 and 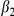 are obtained by the least square estimation with 40 simulation trials data at each time step.

## Supporting Information

Figure S1Changes in the stability of the simplified multistable attractor network model. In each panel, each axis indicates the summed synaptic activity in a subnetwork. The colored curves indicate the nullclines that satisfy the requirement that the time differential of variables on each axis be zero. Namely, in the upper panels with the characteristic axes of the goal representation, the green and orange curves satisfy 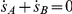 and 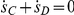, respectively. In the lower panels with the characteristic axes of the action representation, the cyan and purple curves satisfy 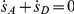 and 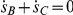, respectively. The gray arrows indicate the vector fields. The closed and open circles at the intersection of the nullclines indicate stable and unstable equilibriums, respectively. In the left panels, the dynamics on the goal-representation axes is bistable (the goal-representation mode). On the other hand, in the right panels, the dynamics on the action-representation axes is bistable (the action-representation mode). See Text S1 for details on the simplified model.(PDF)

Figure S2 Possible network structure that performs higher-dimensional representational switching. The model shown in the main text is a simplified version of these networks. (A) A minimal model that performs representational switching among four goals and four actions. (B) A generalized model that performs representational switching among many more fragments of information belonging to different categories of information, and a neuron may be shared by more than two cell assemblies.
(PDF)

Figure S3 Simulation results in networks consisting of a single type of short-term plasticity. Each panel is in the same format as Figure 3 in the main text. The networks consist of a single type of short-term plasticity with either depression synapses (A–C) or facilitation synapses (D–F). Details of the network structure are described in Methods in the main text.
(PDF)

Figure S4 A schematic view of the possible learning mechanisms of the functional network. (A) In the early stage of learning, neurons are randomly connected with homogeneous distribution of facilitation and depression synapses (blue and red dotted lines, respectively) and with a diversity of synaptic weights. (B) In the process of learning, when neurons coincidentally exhibit correlated activity (small red circles) and contribute to reward acquisition, the synapses between these neurons are selectively strengthened (blue and red solid lines) (see the text for the description of the mechanism). (C) These learning rules may finally lead to a functional network with inhomogeneous connectivity.
(PDF)

Text S1 Dimension reduction using principal component analysis, and dynamics of the multistable attractor model on characteristic axes.
(PDF)
