# Supplementary figures and images for: Representational Switching by Dynamical Reorganization of Attractor Structure in a Network Model of the Prefrontal Cortex
Source: PLoS Comput Biol. 2011 Nov 10;7(11):e1002266. doi: 10.1371/journal.pcbi.1002266 (PMC3213170; doi:10.1371/journal.pcbi.1002266)

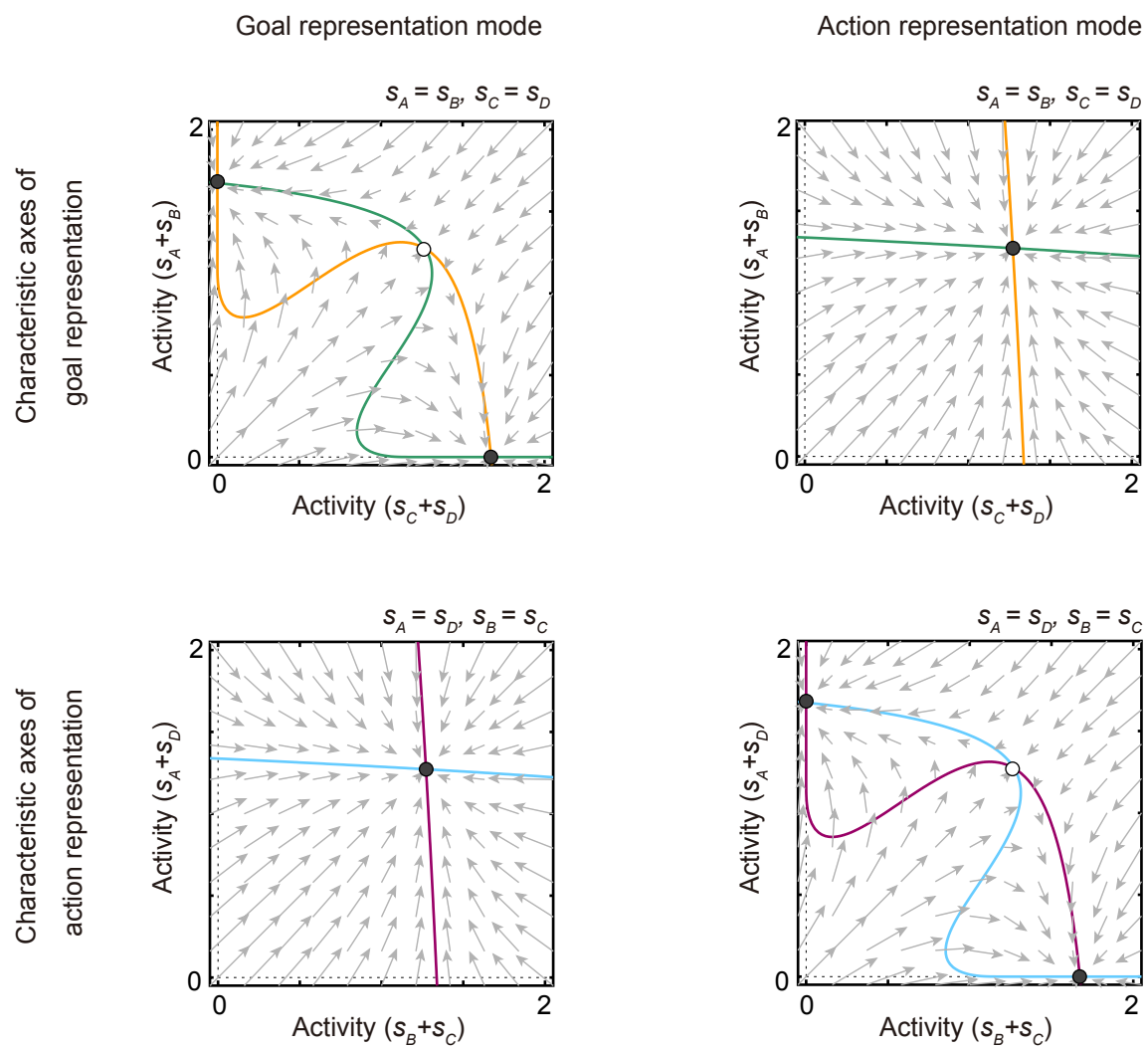

Figure S1

Supplement: Figure S1 — Changes in the stability of the simplified multistable attractor network model. In each panel, each axis indicates the summed synaptic activity in a subnetwork. The colored curves indicate the nullclines that satisfy the requirement that the time differential of variables on each axis be zero. Namely, in the upper panels with the characteristic axes of the goal representation, the green and orange curves satisfy and , respectively. In the lower panels with the characteristic axes of the action representation, the cyan and purple curves satisfy and , respectively. The gray arrows indicate the vector fields. The closed and open circles at the intersection of the nullclines indicate stable and unstable equilibriums, respectively. In the left panels, the dynamics on the goal-representation axes is bistable (the goal-representation mode). On the other hand, in the right panels, the dynamics on the action-representation axes is bistable (the action-representation mode). See Text S1 for details on the simplified model. (PDF) [file pcbi.1002266.s001.pdf]

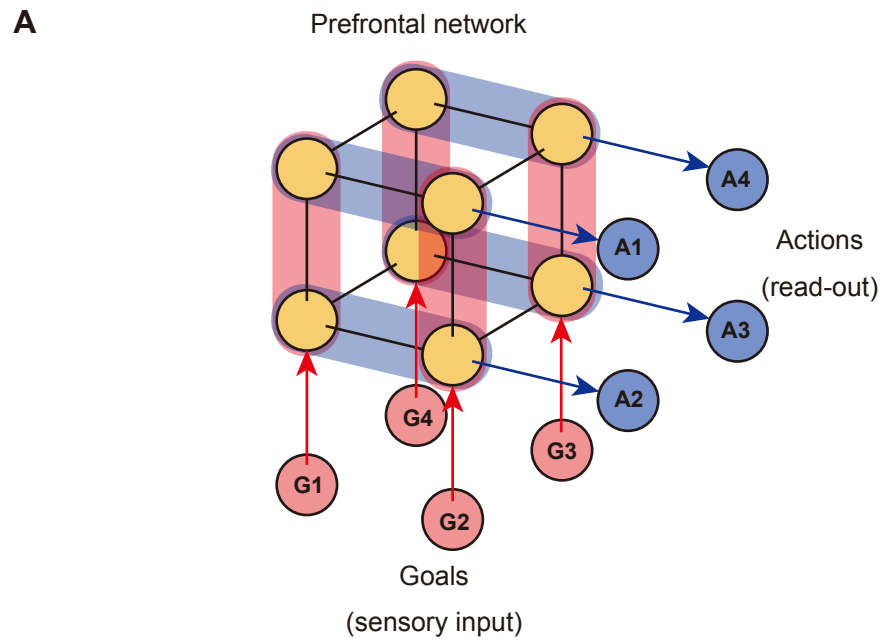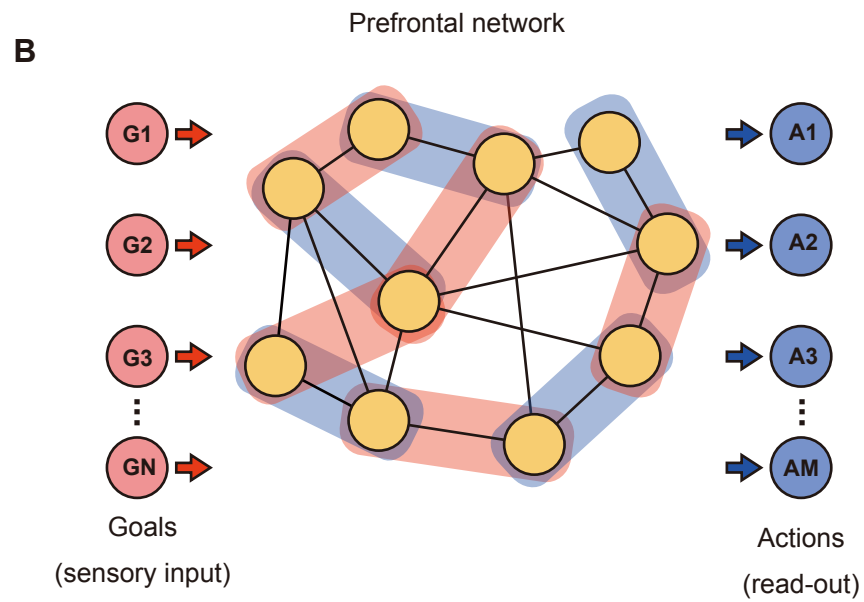

Figure S2

Supplement: Figure S2 — Possible network structure that performs higher-dimensional representational switching. The model shown in the main text is a simplified version of these networks. (A) A minimal model that performs representational switching among four goals and four actions. (B) A generalized model that performs representational switching among many more fragments of information belonging to different categories of information, and a neuron may be shared by more than two cell assemblies. (PDF) [file pcbi.1002266.s002.pdf]

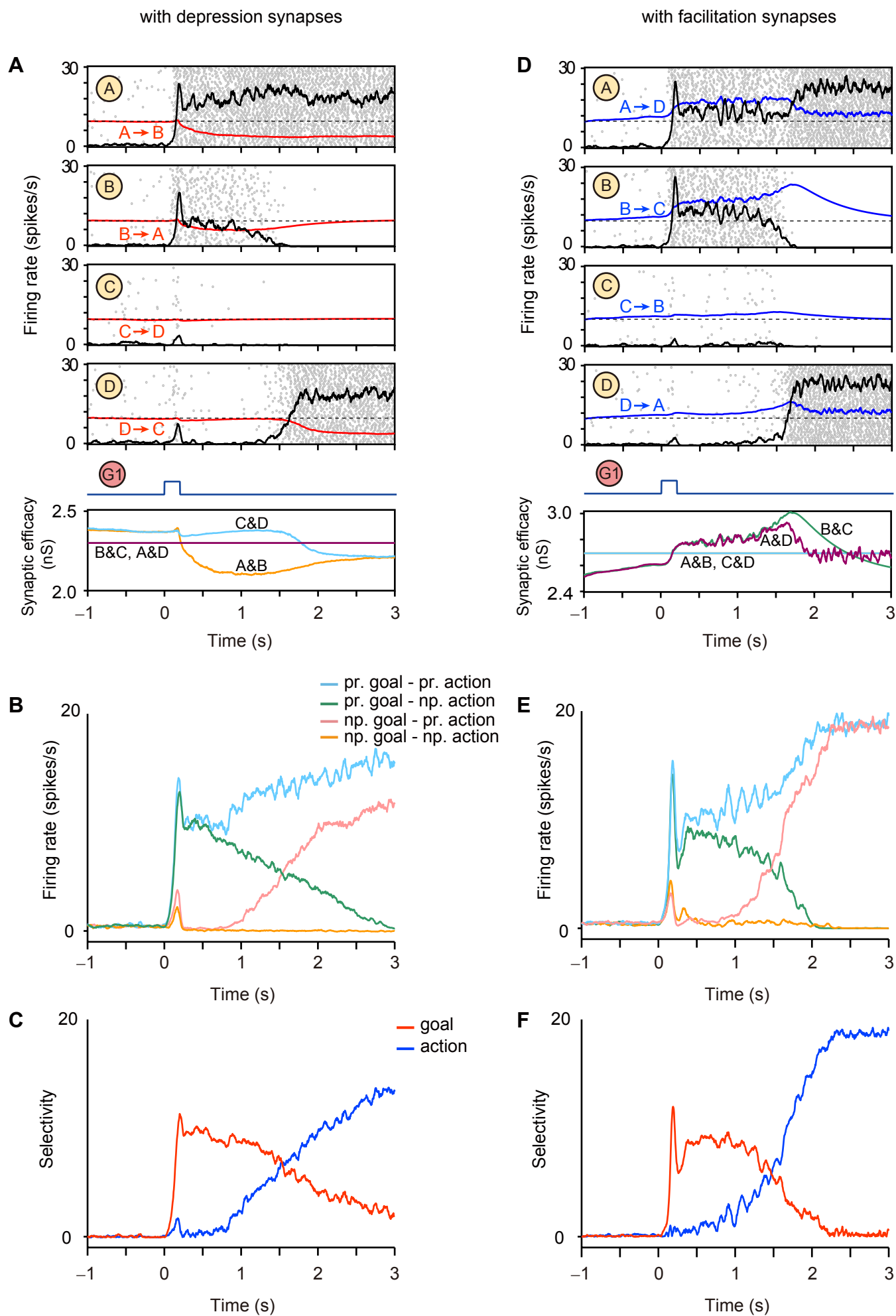

Figure S3

Supplement: Figure S3 — Simulation results in networks consisting of a single type of short-term plasticity. Each panel is in the same format as Figure 3 in the main text. The networks consist of a single type of short-term plasticity with either depression synapses (A–C) or facilitation synapses (D–F). Details of the network structure are described in Methods in the main text. (PDF) [file pcbi.1002266.s003.pdf]

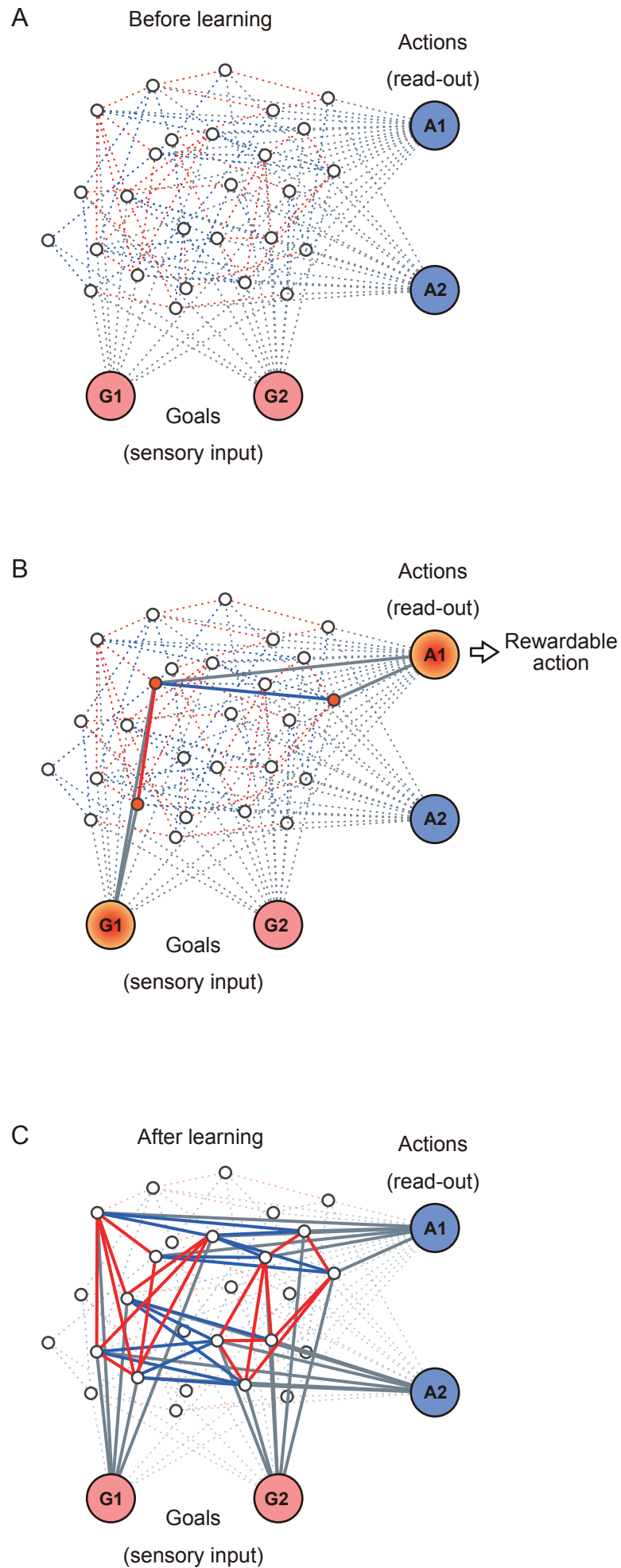

Supplement: Figure S4 — A schematic view of the possible learning mechanisms of the functional network. (A) In the early stage of learning, neurons are randomly connected with homogeneous distribution of facilitation and depression synapses (blue and red dotted lines, respectively) and with a diversity of synaptic weights. (B) In the process of learning, when neurons coincidentally exhibit correlated activity (small red circles) and contribute to reward acquisition, the synapses between these neurons are selectively strengthened (blue and red solid lines) (see the text for the description of the mechanism). (C) These learning rules may finally lead to a functional network with inhomogeneous connectivity. (PDF) [file pcbi.1002266.s004.pdf]
