# Supplementary material for: Representational Switching by Dynamical Reorganization of Attractor Structure in a Network Model of the Prefrontal Cortex
Source: PLoS Comput Biol. 2011 Nov 10;7(11):e1002266. doi: 10.1371/journal.pcbi.1002266 (PMC3213170; doi:10.1371/journal.pcbi.1002266)
Supplement: Text S1 — Dimension reduction using principal component analysis, and dynamics of the multistable attractor model on characteristic axes. (PDF) [file pcbi.1002266.s005.pdf]

Supporting Information

Representational Switching by Dynamical Reorganization of Attractor Structure in a Network Model of the Prefrontal Cortex

Yuichi Katori, Kazuhiro Sakamoto, Naohiro Saito, Jun Tanji, Hajime Mushiake, and Kazuyuki Aihara

Supplementary Methods

Dimension Reduction Using Principal Component Analysis

Using principal component analysis (PCA) [1], the correlated activity of neuron populations can be transformed to an uncorrelated smaller number of variables. We applied this method to the multi-trial time courses of four excitatory neural populations: the synaptic activity of the spiking neuron model (the average of $s_{k,i}^{L}$ in each population) and that of the mean field model. In both models, the results of PCA are similar, and the PCs are almost proportional to the difference between the summed activity of neurons in one subnetwork and that in another subnetwork. Namely, the first PC representing goals were almost proportional to $(s_A + s_B) - (s_C + s_D)$, and the second PC representing actions were almost proportional to $(s_A + s_D) - (s_B + s_C)$. In the present study, these PCs were adopted as the characteristic axes of goal and action representations. The time of the state transition is defined as the first time after the Go signal onset that the absolute value of the action representing second PC exceeds that of the goal representing first PC.

Dynamics of the Multistable Attractor Model on Characteristic Axes

The dynamics on the characteristic axes in Figure 2B of the main text is based on phase-

plane analysis of the mean field model (Figure S1). We analyzed the dynamics on characteristic axes by considering the following simplification of the mean field model. First, the characteristics of the response function of the inhibitory interneurons are approximated by a summation of the input conductances from the populations of the excitatory neurons. The inhibitory interneurons send inhibitory inputs to the excitatory neurons, and the inhibitory conductance is given by $g_{IN}^{I} = G \cdot s_{IN}^{I} = G \cdot (s_A + s_B + s_C + s_D)$, where G = 3.6 nS. Then, the four variables of synaptic activity on the populations of excitatory neurons are reduced to two-dimensional dynamics according to the characteristic axes. The characteristic axes representing goals and actions can be defined by the difference of the summed activity values of the cell assemblies that encode fragments of information. Namely, the characteristic axis of goals (actions) can be defined as the difference between the summed activity of population A and B and that of C and D (the summed activity of population A and D and that of B and C).

Figure S1 shows the dynamics of the simplified model on a phase plane, the axes of which are the summed activity values. In the goal-representation mode (Figure S1, left), the dynamics on the goal-representation axes (Figure S1, upper) is bistable, whereas the dynamics on the action-representation axes (Figure S1, lower) is monostable. In the action-representation mode after the representational switching (Figure S1, right), the stability on the two kinds of characteristic axes is switched. The dynamics on the goal-representation characteristic axes becomes monostable (Figure S1, upper right) and that on the action-representation characteristic axes becomes bistable (Figure S1, lower right). The phase-plane analysis of the simplified model were performed using XPPAUT [2].

References

1. Jolliffe I (2002) Principal component analysis: Springer-Verlag, New York,.
2. Ermentrout B (1990) Phase plane: the dynamical systems tool. Pacific Grove, CA: Brooks/Cole.
